# Factors associated with orthodontic pain

**DOI:** 10.1111/joor.13227

**Published:** 2021-08-01

**Authors:** Wei Lin, Mauro Farella, Joseph S. Antoun, Ruth K. Topless, Tony R. Merriman, Ambra Michelotti

**Affiliations:** ^1^ Discipline of Orthodontics Sir John Walsh Research Institute University of Otago Dunedin New Zealand; ^2^ Department of Surgical Sciences University of Cagliari Sardinia Italy; ^3^ Department of Biochemistry Faculty of Dentistry University of Otago Dunedin New Zealand; ^4^ Department of Neuroscience, Reproductive Science and Oral Science University of Naples Federico II Naples Italy

**Keywords:** anxiety, catastrophising, ecological momentary assessment, genetic polymorphisms, orthodontics, pain

## Abstract

**Background:**

Pain experienced at teeth during orthodontic treatment varies largely over time, with the reasons for its interindividual variability being largely unknown: age, sex, clinical activations, psychosocial factors and genetic polymorphisms of candidate genes are putative factors that may account to explain this variability. We aimed to investigate the effect of clinical, demographic, psychological and genetic factors on pain levels experienced during fixed orthodontic treatment.

**Methods:**

A convenience sample of 183 patients undergoing full‐fixed orthodontic treatment were recruited. Participant's pain levels were assessed seven times over a three‐day period via a smartphone app. Clinical, demographic and psychological data were collected via questionnaires. This included the Pain Catastrophising Scale (Child version), the Corah Dental Anxiety Scale and the State and Trait Anxiety Inventory. Participants provided a DNA sample either in the form of blood or saliva, which were used for genotyping COMT gene rs6269, rs4680, rs4646310, NR3C1 gene rs2963155 and the HTR2A gene rs9316233.

**Results:**

Bond ups had the greatest influence on perceived levels of pain experienced on teeth during orthodontic treatment, accounting for over 20% of total variance in pain response. High‐pain responders had higher scores on pain catastrophising (magnification subscale). Self‐reported pain during fixed orthodontic treatment was not influenced by sex, age, time into treatment, anxiety, nor by polymorphisms of COMT, HTR2A or NR3C1 genes.

**Conclusions:**

Pain on teeth resulting from orthodontic fixed appliances is stronger during bonds‐up and in patients with high catastrophising scores. Demographics, type of clinical activations and the genetic polymorphisms investigated in this research had little or no impact on perceived pain levels.

## Abstract 2

We investigated putative factors influencing orthodontic pain using ecological momentary assessment in 183 study participants. The findings suggest that bond ups had the greatest influence on perceived levels of pain experienced on teeth during orthodontic treatment, accounting for over 20% of total variance in pain response. High‐pain responders had higher scores on pain catastrophising (magnification subscale). Self‐reported pain during fixed orthodontic treatment was not influenced by sex, age, time into treatment, anxiety, nor by polymorphisms of COMT, HTR2A or NR3C1genes.

## 1 INTRODUCTION

Pain at teeth is commonly experienced during fixed orthodontic treatment^1^ and is associated with poor compliance,^2^ a temporary change in diet,^3^ sleep disturbances^4^ and in extreme cases, the discontinuation of orthodontic treatment prematurely.^5^

Pain perceived during fixed orthodontic treatment varies widely between individuals, even when a similar stimulus is applied, *for example* same initial wire.^6^ The cause of this wide interindividual variability in pain perception during fixed orthodontic treatment is largely unknown and has been ascribed to the amount of orthodontic forces,^7^ female sex^8^ and age.^9^

It is generally accepted that there is a delayed onset of pain following the placement or adjustment of fixed orthodontic appliances, with a pain‐free period of two to four hours after adjustments.^10^ Pain at teeth has been shown to peak some 24 to 48 hours after orthodontic adjustment appointments, which then gradually decreases and returns to baseline within five to seven days.^4^ This pattern of pain following orthodontic adjustments is consistent with the time course found in a previously published study.^11^

Historically, the intensity of pain was thought to be directly related to the amount of tissue damage,^12^ but this cannot explain the wide range of pain perception. Nowadays, it is commonly accepted that psychological factors may be more important determinants of pain perception.^13^

Dental anxiety is one of the most common fears,^14^ with a prevalence between 10% and 20%.^15^ Dental anxiety has been positively associated with a patient's pain perception during orthodontic treatment,^16^ routine scaling and cleaning,^17^ dental injections^18^ and general dental treatment.^19^

Pain catastrophising is a term used to describe individuals who exaggerate their pain experience more than the average person, or those who have a ‘negative cognitive‐affective response to anticipated or actual pain’.^20^ Pain catastrophisers are individuals who are often highly anxious and worried about the perception of pain, they often magnify the actual pain experience, feel helpless in the presence of painful events and ruminate on past painful experiences. Pain catastrophising has been shown to have a positive correlation with patient‐perceived disabilities following soft tissue injuries^21^ and higher pain experiences during dental hygiene appointments.^17^ Classifying individuals as either non‐catastrophisers or catastrophisers can be difficult as there is no determinant cut‐off score, rather individuals should be assessed on a spectrum with pain catastrophising scores being best treated as a continuous variable as opposed to a categorical variable.^22^

The COMT gene is implicated in pain perception and is found on the long arm of chromosome 22 and codes of the enzyme catecholamine‐O‐Methyltransferase, which is involved in the degradation of catecholamines (eg dopamine, adrenaline and noradrenaline) as well as catechol oestrogens and other substances which have a catechol structure. It has been suggested that multiple Single Nucleotide Polymorphisms (SNPs) have a synergistic effect on COMT enzyme activity, producing a much more pronounced effect than any singular SNP.^23^ Diatchenko et al 2005 separated subjects into three major haplotypes, LPS (low‐pain sensitivity), APS (intermediate pain sensitivity) and HPS (high‐pain sensitivity). SNPs of the COMT gene have been associated with oro‐facial pain as well as differences in pain perception.^24^, ^25^

The HTR2A gene is located on chromosome 13 and codes for a serotonin G protein‐coupled membrane receptor. It is involved in the serotoninergic system with serotonin being the main neurotransmitter. The serotoninergic system has many functions, such as learning and memory, but perhaps is most well known as a contributor to a feeling of well‐being and happiness. Whilst the HTR2A has not been as extensively studied as the COMT gene, certain SNPs has been associated with musculoskeletal pain^26^ and temporomandibular joint disorders.^27^

The NR3C1 gene is located on chromosome 5 and codes for a glucocorticoid receptor. This receptor is involved in the primary endocrine stress axis in humans, which plays an important role in pain perception. Though the role of this gene and its exact function has not been well studied, SNPs of the NR3C1 gene have been associated with chronic fatigue syndrome^28^ and oro‐facial pain.^27^

In a pilot study, we investigated the association between certain genetic polymorphisms and the patient's pain experience during fixed orthodontic treatment.^29^ The preliminary findings indicated that individuals with the genotype AA of the COMT gene (rs464310) and CG of the HTR2A gene (rs93116233) could experience significantly more pain during fixed orthodontic treatment.

We hypothesised that patient's self‐reported pain levels during fixed orthodontic treatment are influenced by clinical factors, psychological factors, such as anxiety and pain catastrophising, as well as certain single nucleotide polymorphisms (SNP) of the COMT, HTR2A and NR3C1 genes.

## 2 METHOD

This prospective study was conducted at the Clinic of Orthodontics of the University of Otago, between June 2016 and April 2019, and was designed according to the STROBE guidelines.^30^ The study was approved by the University of Otago Human Ethics Committee (reference H15/124), and a written informed consent was collected by all study participants.

A convenience sample of 183 patients who fulfilled the inclusion and exclusion criteria were recruited. Inclusion criteria were as follows: orthodontic treatment with fixed appliances in at least one arch, and less than 18 years of age. Exclusion criteria were as follows: orthognathic surgery, diagnosis of depressive disorder, chronic pain conditions, use of any neurologically acting medication that could potentially affect pain sensitivity, and active caries or periodontal disease. The patients were treated using either 018” slot Mini Taurus (Rocky Mountain orthodontics, Alexander prescription) or 018” slot Mini Masters (American Orthodontics, Otago University prescription). Malocclusion traits and severity were not recorded, because previous data have shown that malocclusion traits, such as crowding, play no significant role in perceived levels of pain perception.^4^

Eligible participants completed a self‐reported questionnaire, which contained basic demographic information (sex, age and ethnicity), participants also completed a seven‐page questionnaire which included the Pain Catastrophizing scale for Children, the Corah Dental Anxiety Scale and the State‐Trait Inventory for Children. For DNA collection, blood samples were taken in participants who were willing, whilst a saliva sample was taken in participants who were unwilling to provide blood samples. The blood samples included a 10mL EDTA tube whilst 10mL saliva samples were taken with Genotek^TM^ Oragene‐500 kits. All DNA samples were stored in a refrigerator set at 3℃ and transported weekly to Merriman Laboratories (University of Otago) for storage, DNA extraction and genotyping.

Immediately following an orthodontic appointment participants were issued an Android smartphone (Vodafone Smart Prime 6, with a 5” colour display) and shown how to use an Android smartphone application (MyBraces Experience) on the issued phone.

The app was developed as part of this study to record the severity of pain following an orthodontic adjustment appointment. The app included a series of pain surveys, which asked about the participants’ analgesic consumption, resting pain at the teeth and pain at the teeth immediately after chewing a piece of chewing gum twenty times (Wrigley Extra Wrigley, Chicago, Illinois, USA). The pain surveys used did ask whether participants experienced any pain around the temporomandibular joint (TMJ) or headaches.

Participant reported their pain severity on a digital sliding visual analogue scale (VAS) measuring 9.3cm long, and participants were asked to drag a small circular dot (1.5mm in diameter) on the VAS to rate the severity of pain they felt at the time. The VAS was labelled ‘no pain at all’ on the left‐hand side and ‘worst pain imaginable’ on the right side. Each participant was required to fill out the pain survey outline by app seven times, that is at (1) baseline on day one (immediately after an orthodontic adjustment); (2) 8pm on day one; (3) 8am on day two; (4) 8pm on day two; (5) 8am on day three; (6) 8pm on day three; and (7) 8am on day four. The orthodontic adjustment was made between 10am and 4pm of the same day the survey started.

The app sent screen and audio alerts to participants when it was time to complete the pain survey; the app allowed participants to enter data up to three hours before and up to three hours after the specified time; if no data were entered within this time period, a missing score was recorded. When all pain survey sessions were completed, the answers were stored on a local database (SQLite, https://www.sqlite.org/). Once all the sessions were completed, the data were retrieved from the local database and compiled into a single comma‐separated values file. The file was emailed using a JavaMail API to a Google Mail account. If no internet connection was available, the app notified the user to connect to the internet, and the email was sent.

Adjustment details at the orthodontic adjustment appointments were recorded and coded into five mutually exclusive categories as follows: 1. no arch wires changed +/‐ minor bends in arch wire; 2. one arch wire changed; 3. two arch wire changed; 4. power chain replacement +/‐ minor bends placed in arch wire; and 5. new bond up in at least one arch. Participants were classified into one category only, for example if a participant had two arch wire changed, they were categorised into two arch wire changed only and not into the one arch wire changed group.

Participants were allocated into three groups based on their pain integral scores; (1) High‐pain responders; (2) Average pain responders; and (3) Low‐pain responders. The three groups were determined by summing each participant's pain scores at rest over the seven pain survey sessions they completed on the app. Participants above the 90^th^ percentile were allocated to the high‐pain responders group, participants below the 10^th^ percentile were allocated to the low‐pain responders group, whilst the remaining participants were allocated to the average pain responders group.

Pain catastrophising scores were tallied as an overall score as well as dividing it into its subscales; rumination, magnification and helplessness from the Pain Catastrophising Scale (child version) questionnaire.^31^

State and Trait anxiety scores were tallied from the ‘How‐I‐Feel’ Questionnaire (child version).^32^ Dental anxiety scores were tallied from the Corah Dental Anxiety.^33^

### 2.1 Genotyping

DNA extraction and genotyping were carried out by the team at Merriman laboratories, Department of Biochemistry, University of Otago. DNA was extracted using a chloroform process with an ethanol precipitation on whole blood cells or buccal cheek cells in the instance of saliva samples. The five SNPs from the three candidate genes COMT (rs4680, rs6269, rs464310), HTR2A (rs9316233) and NR3C1 (rs2963155) were genotyped for every participant. This genotyping was replicated in 10% of the sample for quality control check purposes. The 5 SNPs were tested for Hardy‐Weinberg equilibrium. After correcting for multiple testing, all SNPs were in Hardy‐Weinberg equilibrium (Pc >0.15). Genotyping was completed using TaqMan SNP genotyping assays (Thermo Fisher; 5 Carribean Dv, Scoresby, VIC 3179, Australia) along with KAPA ProbeFaster Master mix on Lightcycler 480 real‐time PCA machine. Haplotypes of the COMT gene were phased using PLINK 1.9 from the SNPs rs4680 and rs6269.

### 2.2 Outcomes

The primary outcome measurements were pain at the teeth at rest and after chewing gum in the 72 hours following an orthodontic adjustment. The collected VAS scores were expressed as percentages (0–100). Because VAS scores were not normally distributed, the cumulative score of pain levels assessed across the three days was calculated and defined henceforth in this manuscript as pain integral. This was compared against a participant details of adjustment, their psychological scores (Pain catastrophising, rumination, magnification, helplessness, A‐State and A‐Trait scores and Dental Anxiety scores), and their genotypes and haplotypes.

### 2.3 Statistical analysis

Analysis was complete using Statistical Package for the Social Sciences (SPSS) software (version 25, IBM, NY, USA). Data were analysed using conventional descriptive methods. Preliminary analyses entailed normality tests and tests for equality of variances—the assumption of normal distribution was tested using a one sample Kolmogorov‐Smirnov test. Friedman analysis of variance was used to test the effects of time (seven time points over 72 hours) on the two VAS variables (‘current pain at teeth’ and ‘pain at teeth after chewing’). The square root of the pain integral values of current pain at teeth and pain at teeth after chewing were normally distributed and entered as dependent variables in a multiple regression model, with age, sex, details of orthodontic adjustment and time in treatment as covariates. Psychological traits were analysed using general linear model (one‐way ANOVA), with the psychometric variables as dependent variables, after adjusting the analysis for possible confounders (*ie* age, sex and time in treatment), and using pain profiles as predictor. Pain profiles were categorised using extreme values of pain integral (high pain, average pain and low pain).

Type I error was set at 0.05 and the proportion of explained variance (R^2^) was calculated for each factor. Comparison of means was performed using Student's t test if the data were normally distributed, or using the Mann‐Whitney U test if the data were not normally distributed. Comparisons of proportions and the chi‐square test. One‐Way ANOVA was used to assess differences in pain severity across genotype and haplotypes.

## 3 RESULTS

### 3.1 Sample

A total of 183 patients were recruited for this study, the mean age of participants was 14.9 years (SD 1.5 years) and ranged between 11.9 and 18.6 years. There were a greater number of female participants compared with male participants (females =53%). One‐hundred and sixty (87.9%) participants identified themselves as NZ‐European, nine (4.9%) participants identified as being Maori/Pacific Islander, eight (4.4%) participants identified as being Asian and five (2.7%) participants were categorised to other. The mean time that participants spent in orthodontic treatment prior to being recruited for this study was 7.0 months (SD =7.3 months), with a range from 0 to 41.4 months. Participants who were recruited for this study on the day of their bond up were recorded as 0 months into treatment. The vast majority of participants had fixed appliances in both arches (93.4%), whilst 6.6% only had fixed appliances in one arch.

Overall, 6.9% of pain surveys were not completed by the participants. Missing data were handled by substituting the missing pain score with that from the previous pain survey, based on the principle of the last observation carried forward.

Pain integral levels recorded over the three‐day research period for at rest, after chewing gum and with maximum pain was not influenced by age (F ≤ 2.1; *p* ≥ 0.15), gender (F ≤ 0.9; *p* ≥ 0.35), ethnicity (F≤1.0, *p*=0.40) or time into treatment (F ≤ 2.0, *p* ≥ 0.16).

### 3.2 Orthodontic adjustment

**FIGURE 1:**
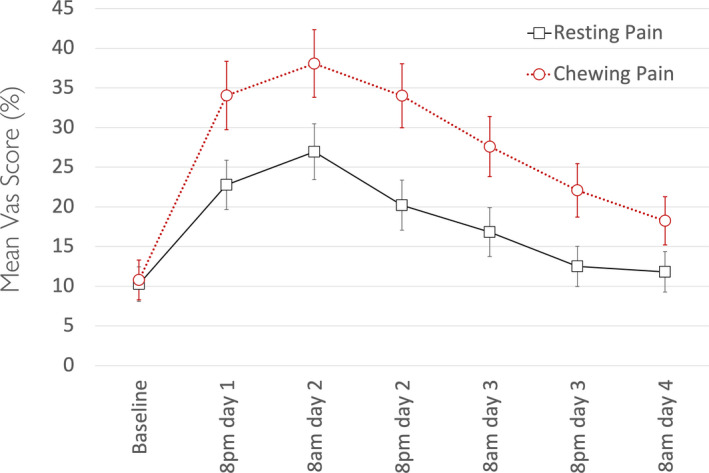
Time‐course profile of VAS ratings for resting pain and chewing pain over the 72 hours. Error bars represent standard deviations. Pain levels peaked the day after the clinical activations and were markedly reduced on day 4

All 183 participants’ data were used for this part of the analysis. The VAS ratings for resting pain and chewing pain over the 72 hours showed a peak the day after the clinical activations and were markedly reduced on day 4 (Figure 1).

**FIGURE 2:**
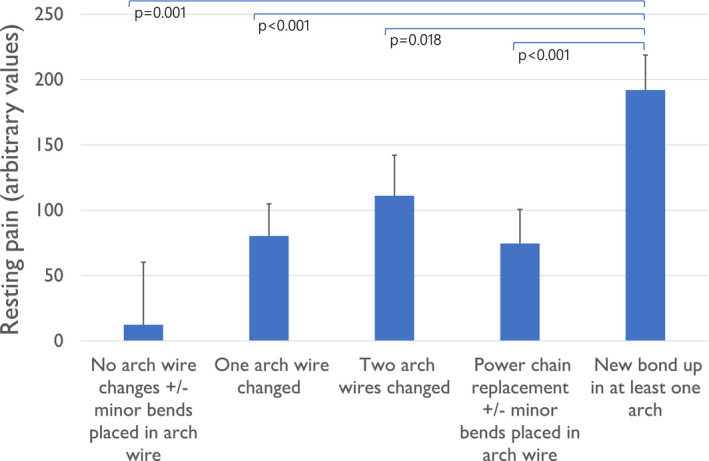
Estimated marginal means of pain experienced at teeth at rest by orthodontic adjustment type. Error bars represent the standard error of mean

**FIGURE 3:**
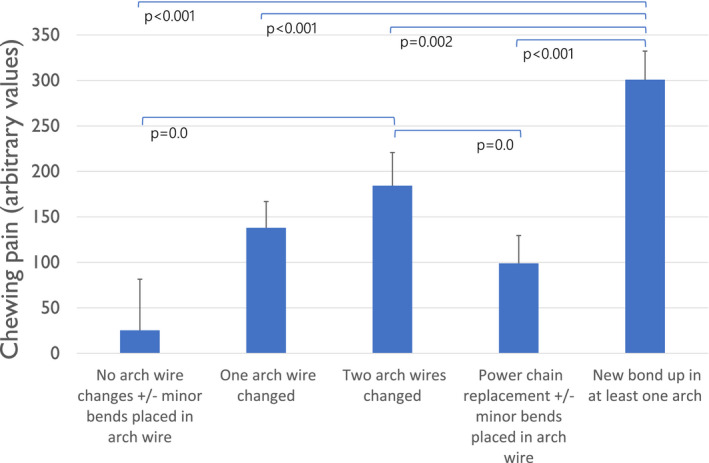
Estimated marginal means of pain experienced at teeth whilst chewing a gum by orthodontic adjustment type. Error bars represent the standard error of mean

Pain integral at rest after a ‘New bond‐up in at least one arch’ was significantly higher compared with all other adjustment types and accounted for about 20% of variance in self‐reported pain scores throughout the study (R^2^ = 0.21) in the multiple regression model, (Figure 2). Pain integral after chewing gum was significantly higher after ‘two arch wire replaced’ when compared to ‘no arch wire change +/‐ minor bends placed in arch wire’ (*p*=0.042) and ‘power chain replacement +/‐ minors bends placed in arch wire’ (*p*=0.039) (Figure 3). The proportion of variance explained by pain after chewing (integral) was over 30% with a R^2^ of 0.31. There were no other statistically significant differences in pain levels when comparing other details of adjustment types. Forty‐five (24.6%) participants reported having a headache during the survey, whilst 12 (6.5%) participants reported having pain in the TMJ area. This pain, however, was consistently reported throughout the survey by only one participant.

### 3.3 Psychological Factors

A total of 177 participants’ data were used for psychological analysis, six participants were excluded due to either missing data or incomplete questionnaires. High‐pain responders showed a pattern to have a higher total pain catastrophising, rumination, helplessness, magnification, A‐State and A‐Trait scores when compared to low‐pain responders. However, only magnification scores were significantly higher the high‐pain responders’ group, when compared to the low‐pain responders’ group (2.8 vs1.4; *p*=0.048).

No statistical difference in the dental anxiety scale score was found between high‐pain responders, average pain responders or low‐pain responders (*p*=0.966). Pain catastrophising, rumination, helplessness, magnification, A‐State, A‐Trait and dental anxiety scale scores did not differ when comparing average pain responders to high‐pain responders nor to low‐pain responders (Table 1).

**TABLE 1:** Mean psychological trait scores across three different pain responder groups

	Pain Profile Group			F Value	P Value
	High Pain (n =18)	Average pain (n=141)	Low Pain (n=18)		
Catastrophising (SEM)	14.2 (1.9)	15.1 (0.6)	10.4 (1.9)	2.7	0.068
Rumination (SEM)	7.0 (0.9)	7.3 (0.3)	5.8 (3.7)	1.5	0.217
Magnification (SEM)	2.6 (0.5)	2.5 (0.2)	1.3 (0.5)	3.4	0.037*
Helplessness (SEM)	4.5 (0.9)	5.2 (0.3)	3.4 (0.8)	2.2	0.114
A‐State (SEM)	29.5 (1.2)	29.3 (0.4)	27.2 (1.2)	1.4	0.247
A‐Trait (SEM)	33.6 (1.8)	33.3 (0.6)	28.8 (1.8)	3.0	0.051
Mean DAS score (SEM)	7.9 (0.7)	7.8 (0.2)	7.5 (0.7)	0.2	0.854

Note

Data represent estimated marginal means and standard error of the mean (SEM) and were controlled for sex, age and time in treatment.

* R‐squared =0.064.

### 3.4 Genetics data

Of the total 179 DNA samples collected, only 172 participants’ data were used for genetic analysis, and eleven were excluded due to missing DNA data or failure of DNA when genotyped. A total of 107 participants (59.2%) provided DNA in the form of blood samples, whilst 72 (40.8%) provided saliva samples. The AA genotype of the COMT gene at position rs4646310 in one study participant was associated with higher pain levels compared with the AG and GG genotypes, but the difference could not be statistically evaluated. No statistically significant difference was found between the remaining SNPs of the COMT, HTR2A and NR3C1 genes (F ≤ 3.0; *p* ≥ 0.05, Table 2).

**TABLE 2:** Resting pain scores (integral) by SNPs

Candidate SNPs/Genotypes	Frequency(%)	Current pain at teeth	Pain after chewing gum
		(Mean ±SEM)	(Mean ±SEM)
rs6269 (COMT)			
AA	70 (40.7)	186.6 ± 44.0	209.5 ± 55.7
AG	84 (48.8)	224.6 ± 52.1	218.1 ± 66.0
GG	18 (10.5)	209.6 ± 62.2	228.9 ± 78.7
rs4680 (COMT)			
AA	54 (33.1)	215.1 ± 61.9	221.2 ± 78.3
AG	79 (48.5)	178.9 ± 47.9	204.1 ± 60.7
GG	30 (18.4)	226.8 ± 46.5	231.2 ± 58.9
rs4646310 (COMT)			
AA	1 (0.6)	374.0 ± 124.0	317.0 ± 157.0
AG	56 (32.9)	109.0 ± 33.8	149.2 ± 42.8
GG	113 (66.5)	137.8 ± 32.7	221.8 ± 75.4
rs2963155 (NR3C1)			
AA	106 (62.4)	190.9 ± 51.4	199.0 ± 65.1
AG	58 (34.1)	208.8 ± 52.9	235.7 ± 67.0
GG	6 (3.5)	221.1 ± 59.6	221.8 ± 75.4
rs9316233 (HTR2A)			
CC	101 (62)	211.7 ± 47.8	214.3 ± 60.5
CG	56 (34.4)	229.4 ± 47.8	256.4 ± 60.5
GG	6 (3.7)	179.7 ± 62.5	185.8 ± 79.2

Note

Data represent estimated marginal means and standard error of the mean (SEM) and were controlled for sex and ethnicity.

## 4 DISCUSSION

After an orthodontic adjustments, our study participants experienced mild‐to‐moderate levels of pain, which steadily increased following the adjustment and peaked at 8am of day 2 and then gradually returned to baseline values by day four. Pain experienced after chewing was consistently higher that resting pain at teeth. These pain profiles are consistent with previous findings 4, 5.

It is a common belief that there is a direct relationship between the amount of force applied to a tooth and the amount of pain a patient would perceive. Though some studies have shown a positive correlation between the two variables,^7^, ^34^ the relationship is still controversial. The results from this study indicate that the introduction of a new appliance has a larger impact on pain perception compared with a change in the force applied. This can be observed where participants with ‘New bond‐ups’ perceived the greatest amount of pain, and it is this variable that accounted for 20% of the overall variance in pain perception across the entire study.

When compared to low‐pain responders (bottom 10%), high‐pain responders (top 10%) tended to have a higher pain catastrophising scores (14.7 vs 11.2) across all subscales (rumination, magnification and helplessness) and have higher A‐State (29.1 vs 27.5) and A‐Trait (34.3 vs 29.7) scores. However, only the magnification subscale scores were significantly different. Though literature has shown that pain catastrophising levels are predictors for patient's self‐reported pain levels^21^, ^35^; however, these studies were not conducted in relation to orthodontic treatment. The lack of significant difference in A‐State and A‐Trait levels between the two pain groups was consistent with a previous study conducted at the University of Otago which also did not find A‐State and A‐Trait scores to differ between high and low‐pain responders after the placement of orthodontic separators.^36^

Dental anxiety scores were almost identical between the high‐ and low‐pain responders, with both groups have a mean DAS score of 7.8. Most literature cite that dental anxiety levels were positively related to the patient's pain experiences during routine dental procedures.^18^, ^37^ However, orthodontic treatment is quite different from routine dental procedures as it does not often require the use of local anaesthetic and/or handpieces, and thus, the use of the original Corah's Dental Anxiety Scale may not be suitable. More recently, a orthodontic version of the Modified Dental Anxiety Scale (MDAS)^38^ has been developed (Roy and Dempster 2018). This modified questionnaire (MDAS‐Ortho) is short and easy to complete, consisting of only five questions. In future studies, the use of MDAS‐Ortho instead of the Corah Dental Anxiety Scale may be more suitable for assessing dental anxiety related to orthodontics. A previous study at the University of Otago has found high‐pain responders to have significantly higher DAS scores compared with low‐pain responders after the placement of separating elastics (mean: 9.4 vs 6.5, *p*=0.043).^36^ It is possible that dental anxiety may have a greater influence on patient pain levels during the initial stages of orthodontic treatment, *for example* placement of separating elastics, compared with participants in this study who have already commenced orthodontic treatment.

The results of this study suggest that pain perceived during fixed orthodontic treatment has a stronger somatosensory component whilst psychological factors have a lower impact. This is supported by the activation ‘new bond ups in at least one arch’ which accounted for 20% of variance of self‐reported pain and was the only activation type consistently associated with higher levels of self‐reported pain. This indicates that pain perceived during fixed orthodontic treatment is mostly related to the physical perception of pain whilst psychological factors such as anxiety and pain catastrophising play a minor role.

The number of participants with minor allele frequencies in this study ranged from 1 (0.6% rs4646310 COMT, AA) to 30 (18.4%, rs4680 COMT, GG). Though the minor allele of the COMT gene (rs6269 GG, rs4680 GG and rs464310 AA) showed a tendency to report higher levels of pain during treatment, the results could not be statistically evaluated. A study participant with the minor allele (AA) for rs4646310 COMT gene reported almost three times the amount of pain integral compared to the GG and AG genotypes; however, this participant had just undergone a full band‐up; a larger sample size will be required to assess if the minor allele for rs4646310 COMT gene does predispose individuals to have a higher pain response during orthodontic treatment.

The minor alleles (G) of the HTR2A and NR3C1 genes have been shown to have a protective effect against oro‐facial pain (OR:0.62 for NR3C1),^27^ which coincides with the patterns found in this study, though no statistically significant findings were found. In total, there were only six participants with the minor allele for either gene, and a larger sample size and thus a larger group of participants with the minor allele may yield significant findings.

This study utilised an ecological momentary assessment approach via a smartphone app to investigate a patient's pain experience with fixed orthodontic appliances over a three‐day period. This method has been previously shown to be promising and effective method of measuring pain during orthodontic treatment.^11^ Utilising an electronic method of self‐reported pain allowed participants to record their pain experiences ‘in real‐time, in real‐world settings, over time and across contexts’.^39^ There are many advantages to utilising this approach: it reduces the risk of recall bias whilst data entry mistakes are minimised, and digital data are also easier to handle, analysis and easier to store.

Pain surveys conducted by participants were carried out at fixed time points and were not relative to the participants’ baseline measures. This may increase the risk of bias as the time difference between the participants’ baseline measures and their following pain surveys will vary from patient to patient. However, these times were chosen for practical reasons to ensure that participants did not have to complete the pain surveys at inconvenient times, for example in the middle of the night or during school hours.

This study used ‘the last observation carried forward’ (LOCF) to replace missing data. In total, 6.9% of the total pain survey was not completed by the participants. The use of LOCF is associated with a high risk of bias,^40^ and the authors acknowledge that this is one of the limitations of this study.

Blood samples are considered the gold standard source of DNA collection and are much preferred for genotyping compared with saliva samples. Compared with saliva samples, DNA collected from blood samples has a greater yield of DNA, and the DNA is more amplifiable and has a higher genotyping cell rate. The reason for this difference is likely due to the sparse cells available for genotyping in saliva samples‐whole blood cells and buccal cheek cells. These whole blood cells and buccal check cells are also bathed in naturally produced enzymes in saliva, which can further damage these already sparse cells. Indeed, 48.4% of the collected saliva samples failed for specific genotypes/SNPs of certain genes, whilst comparatively no blood samples failed when genotyped.

Overall, this study has found that participants perceive the greatest level of pain after an initial bond up, which accounted for 20% of the reported variance. We were unable to find any statistically significant findings between pain catastrophising, A‐State, A‐Trait, Dental anxiety scores or SNPs of the COMT, NR3C1 and HTR2A genes and participants self‐reported pain levels during orthodontic treatment.

A larger sample size will be required to establish a clearer link between genetic markers and pain experienced during orthodontic treatment, and the current analysis for the genetic data should be considered exploratory, due to limited power and a relatively small convenience sample.

In conclusion, pain at teeth during orthodontic treatment with fixed appliances is stronger during bonds‐up and in patients with high catastrophising scores. Sex, age, type of clinical activations and the genetic polymorphisms investigated in this research had little or no impact on perceived pain levels.

## CONFLICT OF INTEREST

The authors have nothing to disclose.

## AUTHOR CONTRIBUTIONS

All the authors listed have made substantial contributions to conception and design, or acquisition of data, or analysis and interpretation of data; have been involved in drafting the manuscript or revising it critically for important intellectual content; gave final approval of the version to be published; and agreed to be accountable for all aspects of the work in ensuring that questions related to the accuracy or integrity of any part of the work are appropriately investigated and resolved.

## Data Availability

The data that support the findings of this study are available from the corresponding author upon reasonable request.
